# Exploring novel approaches in the systemic therapy of low-grade serous carcinoma of the ovary: a literature review

**DOI:** 10.3389/fmed.2024.1366603

**Published:** 2024-05-21

**Authors:** Giovanna Vieira Giannecchini, Jessé Lopes da Silva, Gustavo de Oliveira Bretas, Alexssandra Lima Siqueira dos Santos, Lais Fernandes Rodrigues Baltar, Andreia Cristina de Melo

**Affiliations:** Oncoclínicas&Co – Medica Scientia Innovation Research (MEDSIR), Sao Paulo, Brazil

**Keywords:** ovarian cancer, low-grade serous carcinoma, molecular features, systemic treatment, novel therapeutic options

## Abstract

By presenting a comprehensive analysis of low-grade serous carcinomas (LGSCs), a subset of epithelial ovarian cancers, this review delves into their distinct molecular characteristics, clinicopathological features and systemic therapy options, emphasizing their differences from high-grade serous carcinomas (HGSCs). Notably, LGSCs exhibit prevalent RAS/RAF/MEK/MAPK pathway activation, KRAS and BRAF mutations, and infrequent p53 mutations. While chemotherapy is commonly employed, LGSCs display lower responsiveness compared to HGSCs. Hormone therapy, particularly endocrine maintenance therapy, is explored due to the higher estrogen receptor expression. Novel therapeutic approaches involving CDK4/6 inhibitors, MEK inhibitors, and antiangiogenic agents like bevacizumab are also investigated. Ongoing clinical trials are striving to enhance LGSC treatment strategies, offering valuable insights for future therapeutic advancements in this challenging ovarian cancer subtype.

## 1 Introduction

Ovarian cancer (OC) is the seventh most common cancer in women worldwide (1). In 2020, approximately 314,000 women were diagnosed with this neoplasm and 207,000 died from the disease (2).

Based on the component from which OC originates, it can be classified into epithelial carcinoma, germ cell tumor and sex cord-stromal tumor. The most common are the epithelial carcinomas, which accounts for about 90% of ovarian tumors (1).

Previously regarded as a singular entity, epithelial ovarian cancers (EOCs) are now increasingly acknowledged as a diverse collection of tumors encompassing various histologic subcategories. These subtypes are characterized by their unique immunohistochemical, histopathological, and molecular attributes (3). The main classification of EOCs encompasses five distinct subtypes, namely high-grade serous carcinomas (HGSCs), endometrioid carcinomas, clear-cell carcinomas, mucinous carcinomas, and low-grade serous carcinomas (LGSCs) (4). In contrast to HGSCs, which constitute approximately 70–80% of all malignant ovarian tumors, LGSCs are infrequent and account for less than 5% of cases (5–7). It is important to note that the exact prevalence of LGSC is subject to significant variability due to historical inconsistencies in defining this subgroup (8).

Low-grade serous carcinomas exhibit particularities in clinical behavior, chemo responsiveness and molecular profile (9). As a rare subtype, it is difficult to compare the efficacy of different systemic therapies since there are few randomized trials to establish an evidence-based standard treatment. Consequently, there is no uniform approach and uncertainties regarding the use of current treatments are faced (10).

The aim of this study is to comprehensively examine the landscape of systemic therapy for LGSC, recognizing its distinctive clinicopathological features, molecular profile, and clinical behavioral patterns compared to other ovarian tumor subtypes. In addition to gaining an in-depth understanding of this specific context, the investigation also sought to uncover novel insights that could guide future therapeutic strategies. To accomplish this, a search for relevant literature was conducted utilizing MEDLINE databases, renowned for their comprehensive coverage of healthcare and medical research information. A search on ClinicalTrials.gov to pull up ongoing studies in the field was also performed.

## 2 Pathology

The existing delineation of ovarian serous carcinoma into high-grade or low-grade subgroups relies on the 2004 proposition made by Malpica et al. and the MD Anderson Cancer Center (MDACC) employing a two-tier system (11). This proposes that the classification primarily hinges on nuclear atypia, supplemented by the secondary characteristic of the mitotic rate. Under this binary framework, tumors displaying mild to moderate nuclear atypia and a mitotic index of up to 12 mitoses per 10 high-powered fields are categorized as LGSCs. Subsequent studies have further validated and solidified this binary classification, demonstrating its enhanced reproducibility and prognostic value compared to the previously suggested three-tier grading system, thereby fostering its widespread adoption globally (12–15).

Low-grade serous carcinomas typically present as a uniform population of cuboidal or low-columnar cells, occasionally demonstrating a flattened morphology, and showcasing an amphophilic or lightly eosinophilic cytoplasm (11). Immunohistochemical staining assumes pivotal importance in the pathological assessment. PAX-2 is markedly expressed in 50% of LGSC cases, contrasting with its absence in HGSCs (16). Estrogen receptors (ER) show frequent expression, and in certain cases, progesterone receptors (ProR) and E-cadherin may also exhibit expression (17). Approximately 28% of patients display Her-2/neu expression, while c-kit is positive in 4.5% of cases (18).

Low-grade serous carcinoma can emerge either *de novo* or as a progression from a serous borderline tumor (SBT). The pathogenesis follows a sequential and gradual development, starting from a serous cystadenoma or adenofibroma, progressing to a SBT with invasive or noninvasive implants, and eventually culminating in the formation of LGSC (15). However, there is ongoing controversy regarding the cells’ origin for LGSC. Some researchers propose that LGSC originates from the fallopian tube rather than the ovary (19). According to this theory, the epithelial inclusion glands are more likely of tubal origin due to the invagination of the ovarian surface epithelium with metaplasia (20). The tubal epithelia near the fimbriated end attach to the ovarian surface, facilitated by chronic inflammation, ovulation, and nonovulation-induced disruption of the ovarian surface. The adherent tubal epithelia have the potential to invaginate into the ovarian cortex, forming ovarian epithelial inclusions, which could be the precursor lesions for serous cystadenoma, SBT, and ultimately LGSC. Additionally, a tubal pathway of pathogenesis has been proposed, suggesting that papillary tubal hyperplasia (PTH) serves as the origin for SBT, noninvasive implants, and endosalpingiosis (20). Finally, a non-PTH tubal pathway may also contribute to the development of these lesions, where normal tubal epithelium exfoliates and implants on the peritoneum or ovary (19).

## 3 Molecular fingerprint

In the evolving landscape of oncology, precision and targeted approaches are becoming increasingly vital. Beyond the traditional reliance on histological classification, a thorough understanding of the molecular characteristics of LGSCs is essential for accurate diagnosis and holds significant prognostic and therapeutic relevance (21). Herein, the focus is to describe the molecular alterations in LGSCs and point out their differences from HGSCs. All this knowledge comprehends targetable therapeutic opportunities for clinical studies.

Low-grade serous carcinomas are more likely to have constitutive activation of the RAS/RAF/MEK/MAPK pathway, while HGSC is more likely to have constitutive activation of the PI3K/AKT/mTOR pathway (22–25).

Molecular alterations in the RAS/RAF/MEK/MAPK pathway are highly prevalent in LGSCs. About two-thirds of LGSCs have some molecular alteration in this pathway, and about 50% harbor *KRAS* mutations (26).

*KRAS* mutations in LGSCs vary from 18 to 54% and are the most common genetic alterations in those tumors. These mutations lead to constitutive activation of the RAS pathway, which promotes uncontrolled growth and proliferation (24, 27, 28). However, the prognostic value of *KRAS* mutations seems to be less elucidated than *BRAF* mutations (29).

The mitogen-activated protein kinase (MAPK) pathway is important for some vital cellular functions, such as differentiation, proliferation, survival, autophagy, and apoptosis. *BRAF* mutations result in constitutive activation of this pathway and a downstream activation of kinases, resulting in uncontrolled cellular growth and carcinogenesis (30). In LGSCs, the frequency of these genetic mutations ranges from 2 to 35% and is infrequent in HGSCs. There has been an indication that BRAF mutations, particularly the V600E type, are associated to a favorable outcome in surgically treated patients and are seldom observed in LGSC patients necessitating systemic therapy. Assessing the presence of BRAF mutation in newly diagnosed patients with LGSC histology may aid in identifying those unlikely to progress to a more aggressive histology or advanced disease. Nevertheless, further prospective studies are needed to elucidate the clinical utility of this test (23, 24, 28, 31–33).

In contrast to HGSCs, p53 mutations are uncommon in LGSCs (34, 35). While mutations in BRCA1, BRCA2, and other genes associated with homologous DNA repair are closely linked to HGSCs, they do not appear to play a central role in LGSCs (36).

**FIGURE 1:**
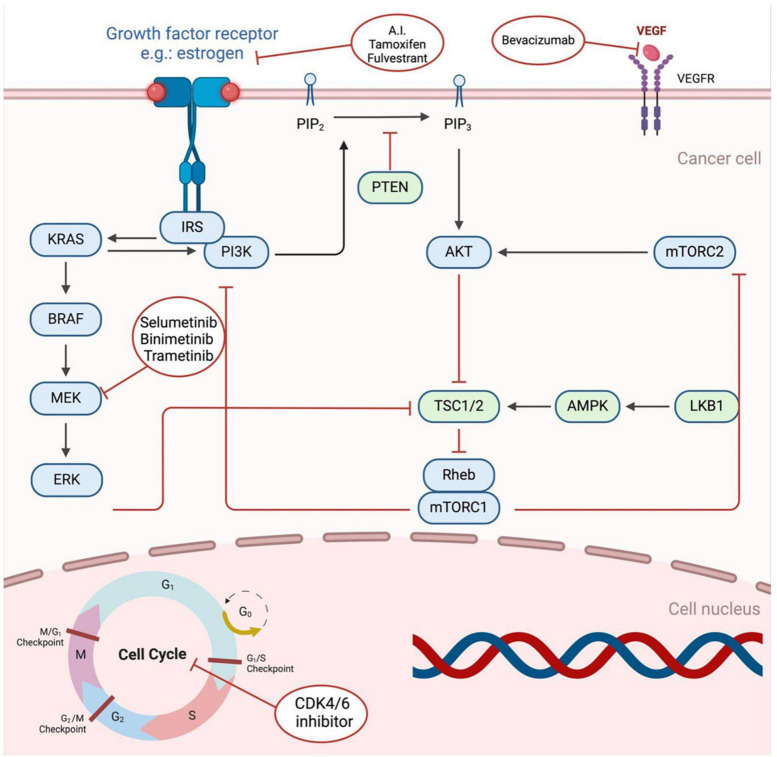
Critical components of the VEGF, Pl3K/AKT/mTOR, and RAS/RAF/MEK signal transduction pathways, as well as the therapeutic interventions employed to target these pathways. Upon ligand binding, the receptors initiate a cascade of signaling events, which are hyperactive in cancerous cells. The diagram provides a visual representation of the key factors involved in these pathways and highlights the therapeutic agents used to intervene in their aberrant activation. Created with BioRender.com.

Figure 1 presents a comprehensive portrayal of the PI3K/AKT/mTOR and RAS/RAF/MEK signaling pathways.

## 4 Surgery

The cornerstone treatment for LGSCs is primary cytoreductive surgery (PCS) (37). An additional examination of the GOG 182 trial, involving 189 patients with FIGO stage III-IV LGSC, demonstrated that achieving optimal cytoreduction (defined as residual disease with a maximal diameter of less than 1.0 cm) was linked to improved median progression-free survival (PFS) and overall survival (OS) in comparison to suboptimal cytoreduction (more than 1.0 cm residual disease): 14.7 months versus 14.1 months for PFS and 44.5 months versus 42.0 months for OS (PFS, *p* < 0.001; OS, *p* < 0.001) (38). While the primary objective is to attain complete gross resection, given the low chemosensitivity of this disease, PCS is advised, even in cases where complete resection may not be attainable (39).

In the context of recurrence, consideration should be given to secondary cytoreductive surgery (SCS). An analysis of 41 patients with recurrent LGSC in a single-institution retrospective study revealed that individuals who directly underwent SCS at the time of progression or recurrence had a longer median OS of 83.3 months, in comparison to 33.2 months for those who initially received systemic therapy (*p* = 0.09) (40). Optimal cytoreduction remains the primary objective for SCS. A systematic review by Goldberg et al. indicated prolonged PFS and OS in patients who underwent complete cytoreduction, and to a lesser extent, optimal cytoreduction, compared to those with suboptimal cytoreduction (41).

## 5 Systemic chemotherapy

Systemic therapy plays a crucial role in the management of LGSC, particularly in cases where the disease has spread beyond the ovaries. Two primary approaches for systemic treatment are chemotherapy and hormone therapy. While these treatments aim to control the progression of cancer and improve outcomes, the choice of the treatment regimen depends on various factors, including the stage, patient characteristics, and individualized treatment plans (42–44).

There is no consensus about the standard adjuvant therapy for stage I LGSC. Routinely, observation is recommended for stage IA disease, whereas stage IB and IC has no universally recognized standardized approach, especially for patients with complete surgical staging (45, 46). Chemotherapy with carboplatin alone for six cycles or carboplatin/paclitaxel for a minimum of three cycles or six cycles if FIGO stage IC, as well as endocrine therapy, are plausible options to be discussed individually with the patients (39).

Following cytoreductive surgery for advanced stages (stage II-IV), patients often receive systemic therapy, which commonly consists of a taxane/platinum regimen (43). This treatment regimen is typically administered every 3 weeks for six cycles and may be succeeded by maintenance endocrine therapy. Intraperitoneal chemotherapy or dose-dense paclitaxel/carboplatin chemotherapy have not demonstrated any established advantages over standard chemotherapy (47, 48).

Despite the widespread use of adjuvant chemotherapy in these patients, studies consistently show the relatively lower responsiveness of this subtype to taxane/platinum chemotherapy compared to HGSCs, with reported response rates ranging from 4 to 23% in newly diagnosed women with stage II to IV LGSCs (49).

For patients with metastatic disease at diagnosis and who are not suitable for surgery, primary systemic therapy with cytotoxic chemotherapy is a feasible option, particularly in those with visceral disease. In such cases, conducting an imaging assessment after three cycles of therapy to identify patients who may benefit from interval cytoreductive surgery is recommended (50, 51).

Regarding neoadjuvant chemotherapy (NACT) for stages III-IV, a retrospective cohort study and systematic literature review with meta-analysis involving less common EOCs was conducted in the United States. 1156 patients with LGSC were included and the use of NACT increased from 7.7 to 14.2% during the study period (*p* = 0.007 for trend) but it was associated with decreased OS compared with PCS (4-year rates, 56.4% versus 81.0%; Hazard Ratio (HR) 2.12; 95% CI, 1.55–2.90) (52).

In a retrospective analysis at Gustave Roussy Institut including 34 patients with LGSC stage IIIb-IV, NACT was administered in 16 patients (47.1%), and complete response (CR) never occurred, which emphasizes the chemoresistance of this disease and the importance of maximum surgical effort (53).

A recently published single-center cohort evaluated 50 patients with LGSC. 58% of the 12 patients with suboptimal residual disease achieved objective responses - 5 partial responses (PRs) and 2 CRs. Only 9% of the 11 patients who had NACT achieved a PR. Overall response rates (ORRs) for platinum-based chemotherapy were 22% in the second line (2 of 9) and 10% in the third line (1 of 10). Primary platinum-based chemotherapy showed moderate activity in LGSC and minimal activity in the recurrent setting, suggesting a need to reconsider platinum sensitivity definitions in LGSC (54).

These findings have highlighted the medical unmet need and the urgency for the development of more efficacious therapeutic approaches for the management of LGSC.

Table 1 provides an overview of published trials on LGSC. Table 2 details the current clinical trials involving LGSC, excluding phase 1 trials and observational studies.

**TABLE 1:** Published trials in low-grade serous ovarian carcinoma.

Study	Intervention	Number of patients	Results	References
Hormonal maintenance therapy for women with low-grade serous cancer of the ovary or peritoneum	__	203	Median PFS = 64.9 months HMT × 26.4 months OBS OS = 115.7 months HMT × 102.7 months OBS	Gershenson et al. (56)
Primary cytoreductive surgery and adjuvant hormonal monotherapy in women with advanced low-grade serous ovarian carcinoma: reducing overtreatment without compromising survival?	__	27	2- and 3-year PFS and OS - 82.8 and 96.3%, and 79.0 and 92.6%, respectively	Fader et al. (57)
Phase 2 trial of ER-positive relapsed ovarian and endometrial cancers	Ribociclib and letrozole	20 patients with ovarian cancer (3 with LGSC)	1 CR and 2 PR, >24 months	Colon-Otero et al. (61)
GOG 0239: A phase II trial of selumetinib in women with recurrent low-grade serous carcinoma of the ovary or peritoneum	Selumetinib	52	OOR = 15% (1 CR, 7 PR, 34 SD)	Farley et al. (63)
MILO/ENGOT-ov11: Binimetinib versus physician’s choice chemotherapy in recurrent or persistent low-grade serous carcinomas of the ovary, fallopian tube or primary peritoneum	Binimetinib	341 (303 patients in the interim analyses)	Median PFS = 9.1 months binimetinib × 10.6 months PCC ORR = 16% Median DOR 8.1 months Median OS = 25.3 months binimetinib × 20.8 months PCC	Monk et al. (64)
Retrospective study of bevacizumab in patients with low-grade serous ovarian and primary peritoneal cancer treated at Memorial Sloan Kettering Cancer Center	__	17 (15 patients received bevacizumab with CT)	PR = 6 patients (all with CT) ORR (15 patients evaluable) = 55% in LGS	Grisham et al. (75)
Retrospective study of bevacizumab containing regimens in recurrent low-grade serous ovarian or peritoneal cancer treated at MD Anderson Cancer Center	__	40	CR = 7.5%; PR = 40% SD = 30%; ORR = 47.5% Median PFS = 10.2 months Median OS = 34.6 months	Dalton et al. (76)
Retrospective study of bevacizumab in low-grade serous ovarian carcinoma	__	12 (11 received Bevacizumab alone)	PR = 8.3% Median PFS = 48 months	Rose et al. (77)
MITO 22 trial: Effect of bevacizumab in advanced low-grade serous ovarian cancer	__	128 (46 LGSC)	Median PFS in first line = 47.8 months with bevacizumab + CT Median PFS in recurrent setting = 37.1 months with bevacizumab + CT	Musacchio et al. (78)
ICON 7: A phase 3 trial of bevacizumab in ovarian cancer	CT + bevacizumab	1528 (80 LGSC)	Mean survival time: 50.4 months with CT × 50.5 months with CT + bevacizumab (HR 0.78 - 95% CI: 0.31– 1.9)	Perren et al. (79)

HMT, hormonal maintenance therapy; OBS, observation; CR, complete response; PR, partial response; OOR, overall response rate; SD, stable disease; PCC, Physician’s Choice Chemotherapy; PFS, progression free survival; OS, overall survival; CT, chemotherapy; LGSC, low grade serous carcinoma; RR, response rate.

**TABLE 2:** Ongoing clinical trials in low-grade serous ovarian carcinoma (excluded phase I and observational trials).

Study	ClinicalTrials.gov ID	Number of patients	Recruitment status
MATAO: Maintenance therapy with aromatase inhibitor in epithelial ovarian cancer	NCT04111978	Estimated enrollment: 540 participants	Recruiting
LEPRE: Letrozole for estrogen/progesterone receptor positive low-grade serous epithelial ovarian cancer	NCT05601700	Estimated enrollment: 132 participants	Recruiting
GOG 3026: a phase II trial of ribociclib plus letrozole in women with recurrent low-grade serous carcinoma of the ovary or peritoneum	NCT03673124	51	Active, not recruiting
A pilot phase II study of fulvestrant plus abemaciclib in women with advanced low-serous carcinoma	NCT03531645	18	Active, not recruiting
GOG 281: Trametinib versus standard of care in patients with recurrent low-grade ovarian cancer or peritoneal cavity cancer	NCT02101788	260	Active, not recruiting
RAMP 201: A study of avutometinib versus avutometinib + defactinib in recurrent low-grade serous ovarian cancer with and without a KRAS mutation	NCT04625270	Estimated enrollment: 184 participants	Active, not recruiting
PERCEPTION: Study of pembrolizumab combination with chemotherapy in platinum-sensitive recurrent low-grade serous ovarian cancer	NCT04575961	Estimated enrollment: 33 participants	Recruiting
Comparison of standard of care treatment with a triplet combination of targeted immunotherapeutic agents	NCT04739800	164	Active, not recruiting
A Study of onapristone ER alone or in combination with anastrozole in gynecologic cancers that respond to progesterone	NCT03909152	34	Active, not recruiting
Letrozole with or without paclitaxel and carboplatin in patients with stage II-IV ovarian, fallopian tube or primary peritoneal cancer	NCT04095364	Estimated enrollment: 450 participants	Recruiting

## 6 Hormone therapy in sequencing systemic treatment

As demonstrated, a very high proportion of LGSCs demonstrate expression of the ER (55). In this scenario, anti-estrogen therapies such as aromatase inhibitors, tamoxifen and fulvestrant are potential treatments.

Therefore, in patients undergoing adjuvant chemotherapy, there might be a potential advantage in utilizing maintenance endocrine therapy, although prospective evidence is required. Gershenson et al. demonstrated in a retrospective study with women diagnosed with stage II to IV LGSCs, that endocrine maintenance therapy after completing primary chemotherapy improved median PFS compared to those who underwent observation alone – 64.9 months (95% CI, 43.5–86.3) versus 26.4 months (95% CI, 21.8–31.0), *p* < 0.001. There was no significant difference in OS, median of 115.7 months versus 102.7 months; *p* = 0.42 (56).

Another approach currently being explored is adjuvant endocrine therapy as a substitute for adjuvant chemotherapy. In a retrospective study, Fader et al. analyzed 27 women with LGSC stage II-IV that underwent cytoreductive surgery followed by endocrine monotherapy, without receiving any adjuvant chemotherapy. Optimal cytoreduction, resulting in the absence of visible residual disease, was achieved in 85.2% of patients. Following a median follow-up period of 41 months, 2- and 3-year PFS and OS were 82.8 and 96.3%, and 79.0 and 92.6%, respectively (57).

There are some ongoing international phase 3 clinical trials evaluating endocrine therapy in women with LGSCs. The MATAO trial (NCT04111978) is recruiting participants to investigate the efficacy of the addition of letrozole in maintenance therapy in women with ER-positive high- and low-grade epithelial ovarian cancer after standard surgery and chemotherapy. LEPRE trial (NCT05601700) is a phase 3 study that is recruiting women with advanced ER- and/or ProR-positive low-grade serous epithelial ovarian cancer to compare letrozole with standard chemotherapy (carboplatin plus paclitaxel). NRG-GY-019 (NCT04095364) is currently enrolling participants and aims to compare the effectiveness of platinum/taxane chemotherapy followed by letrozole maintenance therapy with letrozole monotherapy in women diagnosed with stage II-IV ovarian, fallopian tube or primary peritoneal cancer (including LGSC) who have undergone PCS.

## 7 Cyclin-dependent kinases 4/6 inhibitors

Cyclin-dependent kinases 4/6 (CDK 4/6) play an essential role in regulating cell cycle progression. They bind to the ER-regulated cyclin D1 and mediate the cellular transition from G1 to S phase (58).

In metastatic luminal breast cancer, the role of CDK 4/6 inhibitors in combination with endocrine therapy is well established, with phase 3 studies demonstrating significant improvements in PFS and OS (59). As LGSC presents similarities to hormone receptor-positive breast cancer (60), studies are being developed to evaluate the efficacy of this combination in this scenario.

A phase 2 clinical trial with 40 patients investigated the combination of the CDK4/6 inhibitor ribociclib and the aromatase inhibitor letrozole in the treatment of relapsed ER-positive ovarian and endometrial cancer. Three patients with LGSCs were included and all three obtained durable responses, one CR and two PRs lasting over 2 years (61).

The GOG 3026 (NCT03673124) is a phase 2 trial that evaluated the combination of ribociclib and letrozole in patients with recurrent LGSC. None of the included patients had received prior letrozole or a CDK4/6 inhibitor. The ORR was 23%, with a median duration of response (DOR) of 19.1 months, and 64% of patients experienced a reduction in target lesions. The median PFS was 19.1 months. Such results are promising in a scenario of limited therapeutic options (62).

NCT03531645 is also a phase 2 trial evaluating the role of fulvestrant in combination with abemaciclib in women with advanced LGSCs. This study is still active and will provide important information on the potential of this combination.

## 8 MEK inhibitors

Targeting the MAPK pathway has shown to be a therapeutic opportunity and MEK inhibitors have been evaluated in LGSCs.

Selumetinib, an inhibitor of MEK 1/2, was investigated in a phase 2 study in which 52 women with recurrent LGSC or peritoneal carcinoma were treated until disease progression. The ORR was 15%, with one CR, seven PRs and 34 patients with stable disease (SD) (63).

Other MEK inhibitors have also been evaluated. The MILO/ENGOT-ov11 study was a phase 3 trial that compared binimetinib versus physician’s choice chemotherapy (pegylated liposomal doxorubicin, paclitaxel, or topotecan) in patients with persistent or recurrent LGSC of the ovary, fallopian tube, or primary peritoneum. Median PFS was 9.1 months for binimetinib and 10.6 months for chemotherapy, which resulted in early study closure; however, the MEK inhibitor showed activity in LGSC, with ORR of 16%, median DOR of 8.1 months and median OS of 25.3 months. Patients with *KRAS* mutations had better responses to binimetinib (64).

The GOG 281 is a phase 2/3 trial where patients with recurrent LGSC who had received at least one platinum-based regimen were randomly assigned to receive the oral MEK inhibitor trametinib or the physician’s choice of standard of care therapy (weekly paclitaxel, pegylated liposomal doxorubicin, topotecan, letrozole or tamoxifen). The median PFS was 13 months for trametinib versus 7.2 months for standard of care (HR 0.48; 95% CI 0.36–0.64; *p* < 0.0001). The ORR for the trametinib group was 26%, and a median DOR of 13.6 months versus an ORR of 6% and a median DOR of 5.9 months for standard therapy. Median OS was 37.6 months in the trametinib group and 29.2 months in the standard treatment group, with a HR for death of 0.76 (95% CI 0.51–1.12; *p* 0.056), but the trial allowed cross-over to trametinib after disease progression. Regarding the mutational status, *KRAS*, *BRAF* and *NRAS* mutations were not predictive for PFS. ORR was better for trametinib than the standard of care in mutation-positive than in mutation-negative tumors but not reaching statistical significance (65).

In a multicentre retrospective study in the United Kingdom that included 28 patients with recurrent LGSC treated with trametinib, the median duration of treatment was 5.0 months, with PR, SD, and disease progression (DP) in 21, 32, and 36% of patients, respectively. These real-world response rates were similar to that reported in GOG 281, confirming the effectiveness of these MEK inhibitor in LGSC (66).

## 9 Antiangiogenic agents

Angiogenesis plays a significant role in tumor growth and metastasis, being considered one of the hallmarks of cancer (67). Bevacizumab is a humanized anti-VEGF-A monoclonal antibody and works as an anti-angiogenic drug that is approved in many countries for the treatment of ovarian (mostly high-grade) and other cancers. While bevacizumab has shown benefits in HGSC, its use in LGSC is still a topic of ongoing research and clinical evaluation. Most available evidence comes from subgroup analyses, small retrospective studies, and several case reports (68–74).

The Memorial Sloan Kettering Cancer Center published a series describing patients with recurrent LGSC or serous borderline treated with bevacizumab between 2005 and 2012. Only 17 patients were reported, and most received bevacizumab plus chemotherapy agents. The mean number of prior cytotoxic therapies was 3.4 (range 1–9; median 2). The median duration of bevacizumab administration in evaluable patients was 23 weeks (mean 32.2 weeks; range 6–79.4). There were no CRs, and PRs were observed in 6 patients (5 received concurrent paclitaxel, and 1 received concurrent gemcitabine). The ORR was 40%, with a response rate of 55% amongst the subgroup of patients with LGSCs (75).

The MD Anderson Cancer Center also published their retrospective experience with bevacizumab in recurrent LGSC of the ovary. A total of 40 patients were reported, and the median number of prior regimens was 4 (range 1–15). The average duration of bevacizumab treatment was 4 months, with a range of 0.8–25.4 months. CRs were seen in 7.5% of patients, 40.0% had PRs, while 30.0% achieved SD. DP occurred in 22.5%, and clinical benefit was achieved in 77.5% of patients (76).

The Cleveland Clinic or MetroHealth Medical Center in Cleveland retrospectively published their data on the use of bevacizumab in LGSC. Twelve patients were reported, and 11 of those received bevacizumab alone. The ORR was only 8.3%, but the median PFS was 48 months (range 5–123). The median OS was not reached at a median follow-up of 32 months, with only 1 of the 12 patients dying of disease (77).

In a retrospective observational multicenter study, the authors explored the efficacy of bevacizumab on survival outcomes in women with LGSC both in first-line and recurrent settings, comparing the results with those presented by patients who did not receive bevacizumab. A total of 46 out of 128 patients who received bevacizumab in a first-line setting or at the time of first recurrence were identified. In the first-line, 30 patients received bevacizumab plus chemotherapy and 65 chemotherapy alone, and the median PFS was 47.8 months [95% CI 31.48 to not reached (NR)] and 22.6 months (95% CI 15–39.24), *p* = 0.0392, respectively. In the recurrent setting, 16 patients who received bevacizumab plus chemotherapy were compared to 33 women treated with platinum-based chemotherapy. Median PFS was 37.1 months (95% CI 13.42–40.56) and 11.2 months (95% CI 8.26–15.63), *p* = 0.013, respectively, suggesting that bevacizumab might be an effective drug both in diagnosis and in relapse (78).

In the ICON7 study (79), which investigated the benefits of adding bevacizumab in the first-line setting, only 80 patients with LGSC were included. The addition of bevacizumab resulted in a non-significant HR of 0.78 (95% CI 0.31–1.97; *p* = 0.07) in the sub-analysis (80).

## 10 Conclusion

In conclusion, this thorough review underscores the evolving understanding of LGSCs within the broader context of ovarian cancers. Since it is a rare disease, with few randomized studies, this narrative review was representative in including data on real-world experience, with descriptions of retrospective cohort studies, series of cases, and experience from single centers.

Investigating the distinctive molecular profile, histopathological characteristics, and clinical behavior of LGSC has revealed its unique nature in contrast to HGSC. The higher prevalence of RAS/RAF/MEK/MAPK pathway activation, KRAS and BRAF mutations, and limited p53 involvement provide a foundation for targeted therapies. The challenges in chemotherapy response emphasize the need for innovative treatment strategies. Hormone therapy, mainly endocrine maintenance therapy, offers a promising avenue due to the higher ER expression. Moreover, the investigation into CDK4/6 inhibitors, MEK inhibitors, and antiangiogenic agents unveils potential therapeutic directions for LGSC management. Ongoing clinical trials further highlight these prospects and encourage continued research. Throughout the execution of these studies, the creation of a biorepository for collection and storage of human biological material is essential for ethical purposes and to standardize protocols aiming reliable and reproducible research data.

Finally, the present study humbly underlines the imperative of a multidisciplinary approach to tackle the challenges posed by LGSCs. By dissecting the intricate interplay of molecular characteristics and therapeutic options, this review highlights that the way for more targeted, effective, and personalized treatments should be prioritized in order to improve the prognosis and quality of life for patients with this unique ovarian cancer subtype.

## Author contributions

GG: Investigation, Writing – original draft, Project administration, Writing – review and editing. JS: Writing – review and editing, Investigation, Writing – original draft. GO: Investigation, Writing – original draft, Writing – review and editing. AS: Investigation, Writing – original draft, Writing – review and editing. LB: Investigation, Writing – original draft, Writing – review and editing. AM: Conceptualization, Supervision, Writing – review and editing.
